# Non-Isocyanate Urethane Acrylate Derived from Isophorone Diamine: Synthesis, Characterization and Its Application in 3D Printing

**DOI:** 10.3390/molecules29112639

**Published:** 2024-06-03

**Authors:** Xinqi Zhang, Xinxin Zan, Jiangdi Yin, Jiaxi Wang

**Affiliations:** 1 School of Chemical Engineering and Technology, Hebei University of Technology, Tianjin 300130, China; zxq19692021@163.com (X.Z.); 15716856616@163.com (X.Z.); 18332768376@163.com (J.Y.); 2 Hebei Provincial Key Lab of Green Chemical Technology and High Efficient Energy Saving, Hebei University of Technology, Tianjin 300130, China

**Keywords:** non-isocyanate, urethane acrylate, isophorone diamine, UV curing, 3D printing

## Abstract

In this paper, urethane-based acrylates (UA) were prepared via an environmentally friendly non-isocyanate route. Isophorone diamine (IPDA) reacted with ethylene carbonate (EC), producing carbamate containing amine and hydroxyl groups, which further reacted with neopentyl glycol diacrylate (NPGDA) by aza Michael addition, forming UA. The structures of the obtained intermediates and UA were characterized by ^1^H NMR and electrospray ionization high-resolution mass spectrometry (ESI-HRMS). The photopolymerization kinetics of UA were investigated by infrared spectroscopy. The composite with obtained UA can be UV cured quickly to form a transparent film with a tensile strength of 21 MPa and elongation at break of 16%. After UV curing, the mono-functional urethane acrylate was copolymerized into the cross-linked network in the form of side chains. The hydroxyl and carbamate bonds on the side chains have high mobility, which make them easy to form stronger dynamic hydrogen bonds during the tensile process, giving the material a higher tensile strength and elongation at break. Therefore, the hydrogen bonding model of a cross-linked network is proposed. The composite with UA can be 3D printed into models.

## 1. Introduction

UV curing technology is widely used in many fields such as coatings [1,2,3,4], adhesives [5,6], inks [7,8,9], 3D printing [10,11,12] and many other fields [13,14] due to its advantages of a fast curing rate, low energy consumption and environmental protection. Oligomers are the main components of the UV curing system and have an important impact on the properties of cured materials [15,16,17]. Common UV curing resins are polyurethane acrylates (PUA), epoxy acrylates, polyester acrylates, etc. Polyurethane acrylate not only has the good abrasion resistance and flexibility of polyurethane but also has the excellent chemical resistance and weather resistance of acrylate [18]. The presence of urethane bonds in polyurethane leads to the formation of intermolecular and intramolecular hydrogen bonds, provides many physical cross-linking points and results in the excellent tensile properties of the product [19]. PUAs are generally synthesized by the reaction of NCO-terminated polyurethane and acrylate-terminated alcohol [20,21,22] or polyurethane alcohol with (meth)acrylic anhydride [23,24] and (meth) acryloyl chloride [25,26]. Isocyanate, phosgene and amines, the raw materials for the preparation of isocyanate, are highly toxic compounds [27,28]. In addition, isocyanates are also water-sensitive substances and require water-free operation. Using acrylic anhydride or acryloyl chloride as an acrylate resource, the acryloylation process produces by-products such as carboxylic acid or hydrogen chloride, which are generally not directly used for UV curing and are complex to separate. In terms of safety, the synthesis process of environmentally friendly non-isocyanate polyurethanes (NIPUs) has attracted widespread attention. The most popular reaction for NIPUs preparation is the reaction of multifunctional carbonates with primary diamines [29,30,31,32], amino alcohols [33,34] or polyamines [35,36,37]. There are few studies on the preparation process of non-isocyanate urethane acrylate without using (meth)acryloyl chloride or (meth)acrylic anhydride as raw materials.

**Scheme 1:**
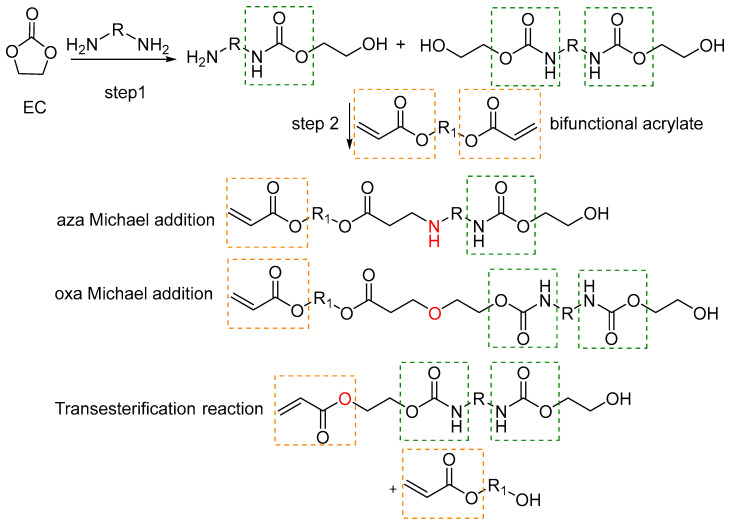
Synthesis route of non-isocyanate urethane acrylates.

Based on the principle of molecular design, without using (meth)acryloyl chloride or (meth)acrylic anhydride as an acryloyl source, an environmentally friendly non-isocyanate polyurethane acrylate synthesis process is proposed as shown in Scheme 1. Step one is to prepare intermediates containing carbamate fragments by the reaction of diamine with EC. Step two is to prepare urethane acrylates using Michael addition and/or the transesterification of intermediates with acrylate. In this work, the Michael addition of alcohol and/or amine with acrylate was investigated. The product structure was characterized by ^1^H NMR, ESI-HRMS and FT-IR. The UV curing activity, thermo-mechanical properties of the cured products and its application in 3D printing were investigated.

## 2. Results and Discussion

### 2.1. Synthesis and Characterization of UA

**Figure 1:**
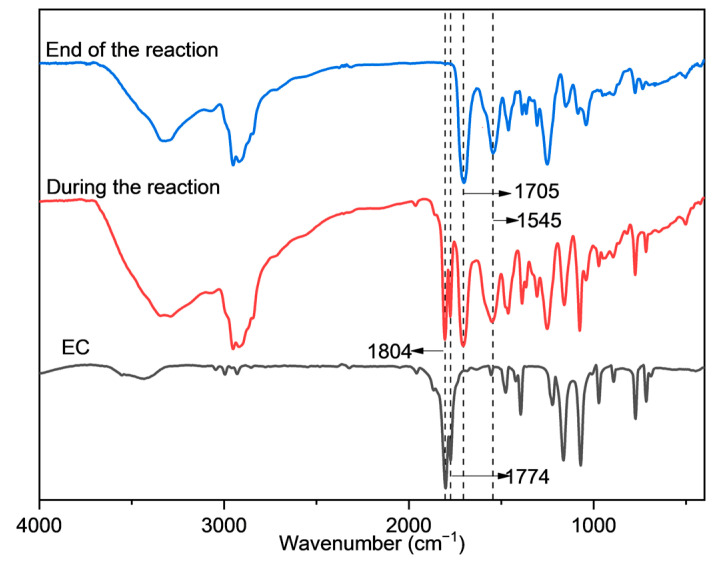
FT-IR spectroscopy monitoring of IPDA and EC reaction processes.

**Figure 2:**
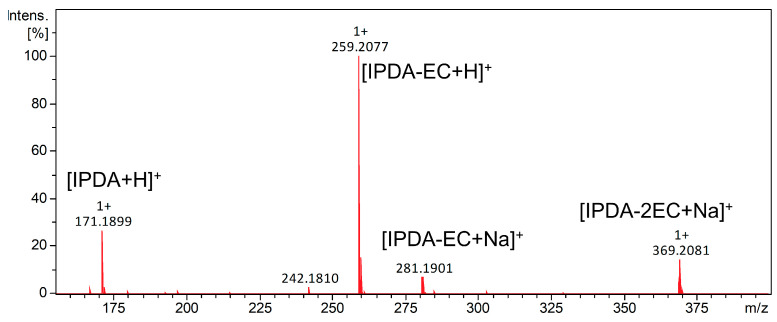
ESI-HRMS spectrum of reaction products of IPDA with EC.

**Scheme 2:**
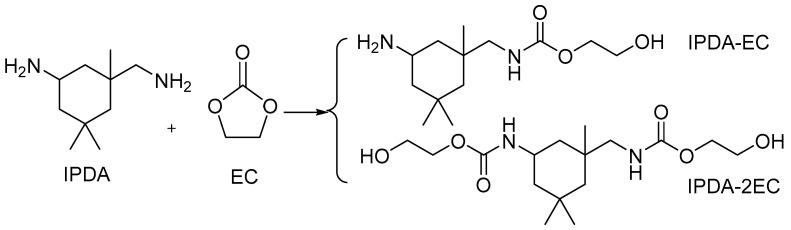
Reaction pathway of IPDA with EC.

Due to the good performance of polyurethane derived from IPDI [38,39], IPDA was used to react with cyclic carbonate to construct non-isocyanate polyurethane fragments [40,41,42]. IPDA contains two amine groups with different chemical environments, and the reactivity of the amine group attached to the secondary carbon atom is low. The carbonyl signal of the EC at 1804 cm^−1^ and 1774 cm^−1^ can be used to monitor the reaction process between IPDA and EC by FT-IR (shown in Figure 1). As the reaction progresses, the carbonyl signal of EC weakens until it disappears, while the bond signals of NH (amide) and carbonyl C=O (amide) increase at 1545 cm^−1^ and 1705 cm^−1^. The ESI-HRMS spectrum of the reaction products of IPDA with EC is shown in Figure 2. As we can see, IPDA reacted with EC at a mole ratio of 1:1, producing 2-hydroxyethyl ((5-amino-1,3,3-trimethylcyclohexyl) methyl) carbamate named as IPDA-EC, along with 2-hydroxyethyl ((5-(((2-hydroxyethoxy) carbonyl) amino)-1,3,3-trimethylcyclohexyl) methyl) carbamate named as IPDA-2EC (shown in Scheme 2). The distribution of IPDA-EC and IPDA-2EC is related to the mole ratio of IPDA with EC. IPDA reacted with two equivalents of EC completely to form IPDA-2EC as the main product (ESI-HRMS is shown in Figure S1, some other product structures are shown in Scheme S1). When the IPDA-2EC was used to react with NPGDA to prepare the UA by the oxa Michael addition or transesterification, we found that the conversion of IPDA-2EC was very low, which indicated that the oxa Michael addition was not an efficient reaction for building the molecular of UA (ESI-HRMS is shown Figure S2). However, the IPDA-EC (minor product from the reaction of IPDA with EC in a mole ratio of 1:2) reacted completely with NPGDA by aza Michael addition, forming IPDA-EC-2NPGDA (the structural formula is shown in Scheme S2), which means that IPDA-EC is a more efficient molecule that combines carbamate with acrylate to form carbamate acrylate. To reduce the formation of IPDA-2EC, EC is added in triplets, and the temperature is increased to 70 °C when a third batch of EC is added (method 1). The ^1^H NMR spectrum of the reaction products of IPDA with EC is shown in Figure S3. The chemical shifts and integrals of all signals are generally consistent with the expected products.

**Figure 3:**
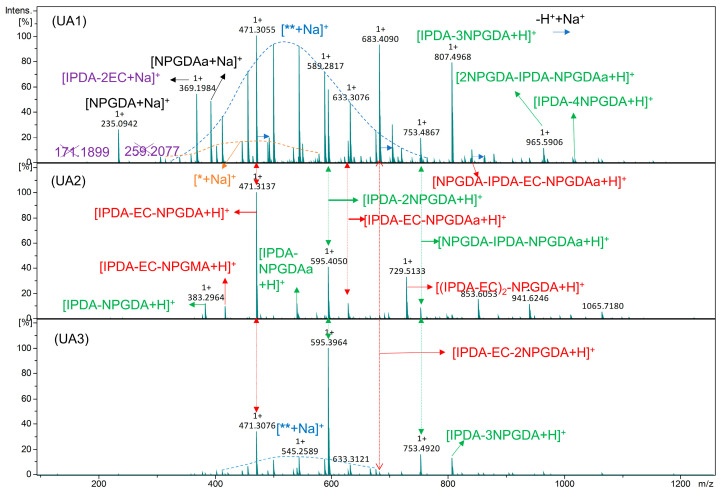
ESI-HRMS spectra of UA1, UA2 and UA3.

**Scheme 3:**
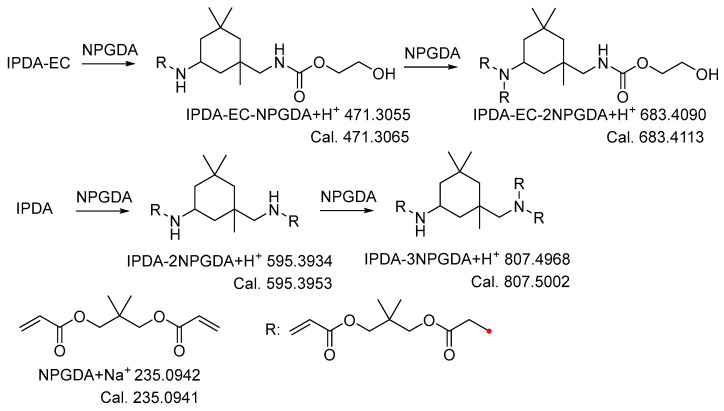
The main structural formula of UA1.

**Scheme 4:**
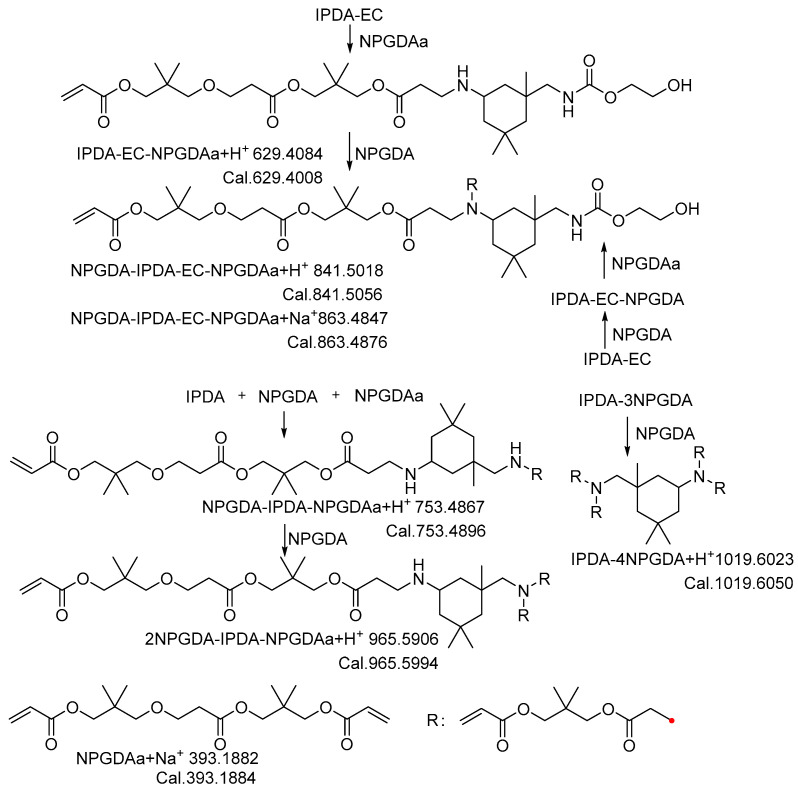
Minor products of UA1.

**Figure 4:**
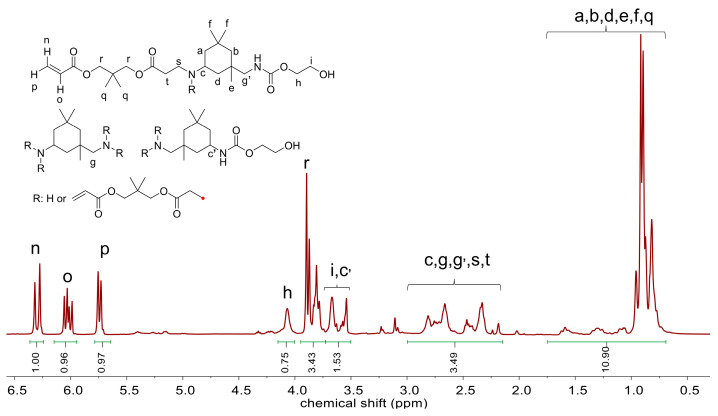
^1^H NMR spectrum of UA1.

The ESI-HRMS spectra of UA1~UA3 are shown in Figure 3. From Figure 3 (UA1), all IPDA-EC and residue IPDA reacted with NPGDA by aza Michael addition, mainly forming IPDA-EC-NPGDA, IPDA-EC-2NPGDA, IPDA-2NPGDA and IPDA-3NPGDA (shown in Scheme 3). Some minor products are shown in Scheme 4. No signal related to the product of IPDA-2EC with NPGDA was observed, indicating that the oxa Michael addition or transesterification are difficult to occur (the signal of IPDA-2EC+Na^+^ is still at 369.1984). Two series of signals marks * and ** are assigned to the acrylates, forming by the transesterification of PEG-like diol with NPGDA (shown in Scheme S3). The PEG-like diol may be formed by the ring-opening reaction of EC catalyzed by tertiary amine. The ^1^H NMR spectrum of UA1 is shown in Figure 4. Based on the analysis of the ^1^H NMR spectrum, the amount of two series acrylates oligomer is about 0.8%. The high signal strength of * and ** acrylates is due to the high responsiveness of PEG-like fragments in ESI-HRMS.

**Scheme 5:**
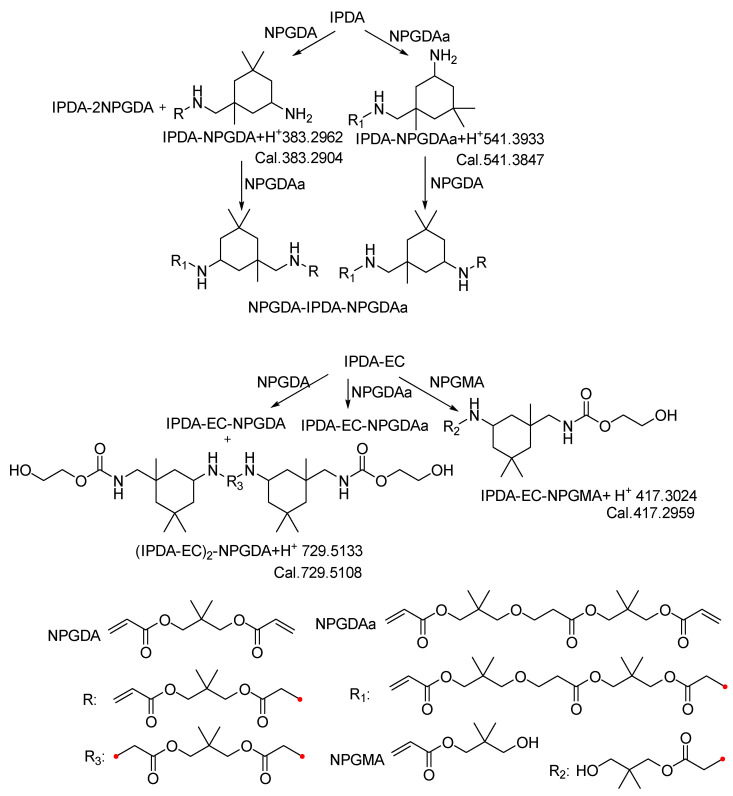
Structural formulas of UA2.

**Figure 5:**
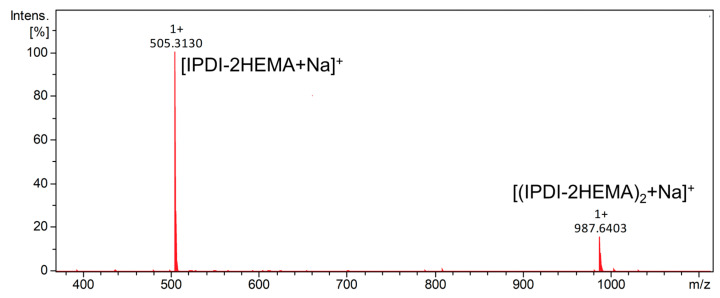
ESI-HRMS spectrum of UA4.

**Scheme 6:**
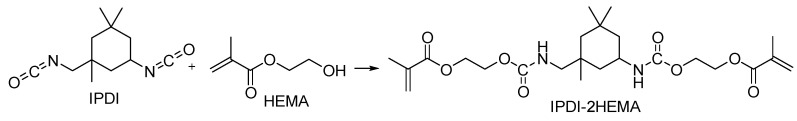
The structure of UA4.

To reduce the viscosity, the third part EC was added with some NPGDA (method 2). From Figure 3 (UA2), the products contain IPDA-EC-NPGDA as the main product, along with some aza Michael addition product of IPDA-EC with other acrylates (IPDA-EC-NPGMA, IPDA-EC-NPGDAa and (IPDA-EC)_2_-NPGDA), and the residual IPDA reacted with acrylates to form acrylate derivatives (IPDA-NPGDA, IPDA-NPGDAa, IPDA-2NPGDA) as by-products (shown in Scheme 5). When the mole ratio of IPDA to EC was 3:2 (method 3), IPDA reacted with EC to form IPDA-EC, which reacted further with NPGDA, forming IPDA-EC-NPGDA and IPDA-EC-2NPGDA. IPDA-2EC was not observed (Figure 3 (UA3)). The remaining IPDA reacted with NPGDA to form IPDA-2NPGDA. Computationally, the remaining IPDA accounts for only one-third of IPDA, so the IPDA-2NPGDA formed is not the main component in the product’s mixture. The IPDA-EC-NPGDA with carbamate group is the main product. Compared to IPDA-EC-NPGDA with one secondary amine group, IPDA-2NPGDA with two secondary amine groups is more likely to capture H^+^ and shows a higher signal on ESI-HRMS. The ^1^H NMR spectra of UA2 and UA3 are shown in Figures S4 and S5. In order to compare the performance of UV cured materials, carbamate UA4 was prepared by reacting IPDI with hydroxyethyl methacrylate by the traditional method. The ESI-HRMS of UA4 is shown in Figure 5. The preparation reaction is shown in Scheme 6.

### 2.2. Photopolymerization Kinetics of UA

**Figure 6:**
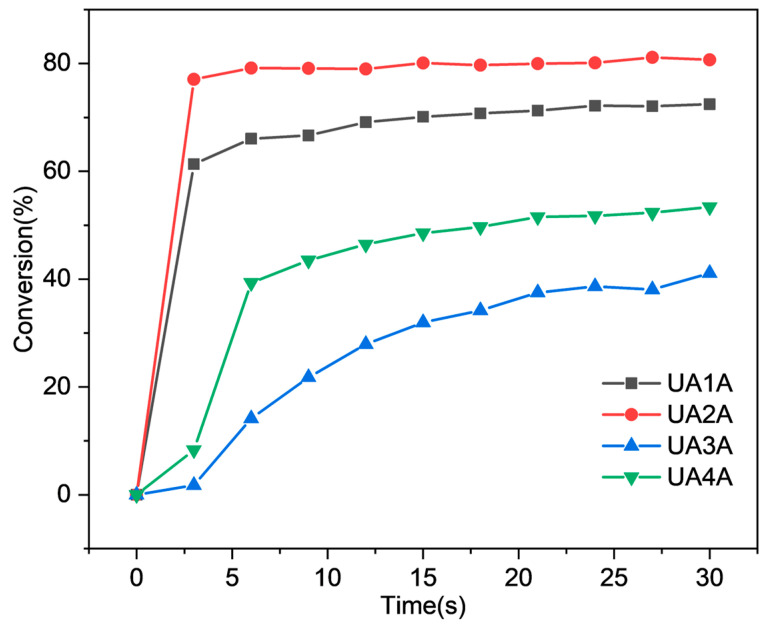
Photopolymerization kinetics of UA1A~UA4A.

The photopolymerization kinetics of UA1A~UA4A were investigated by using FT-IR. The curve of the double bond conversion rate over time is plotted as shown in Figure 6. The FT-IR of photopolymerization kinetics is shown in Figure S6. From Figure 6, the double bond conversion rates of the UA system at 30 s were in the order of UA2A (81%) > UA1A (72%) > UA4A (53%) > UA3A (41%). Since the radical polymerization behavior of methacrylates and acrylates is different, it is inconvenient to compare the UV curing activity of UA4A with UA1A~UA3A. Among the UA1A~UA3A molecules, the relatively high UV curing rate of UA2A may be due to the highest content of acryloyl group (based on ^1^H NMR analysis) and a certain amount of tertiary amine structure. There is a balance between the amount of carbamate acrylate and tertiary amine derived from IPDA. Tertiary amines can be used as chain transfer agents to prolong the lifetime of free radicals and increase the polymerization rate. However, the activity of the free radicals formed after chain transfer by tertiary amine is relatively reduced, resulting in a decrease in the molecular weight and polymerization rate.

### 2.3. Thermal and Mechanical Properties of PUA1A~PUA4A

**Figure 7:**
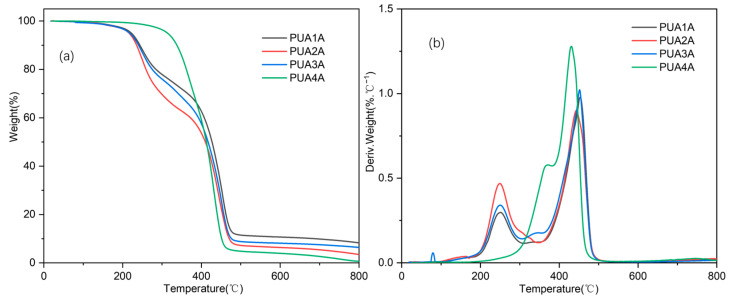
(**a**) TG curves and (**b**) DTG curves of PUA1A~PUA4A.

After UV curing, the cured UA1A~UA4A samples were named as PUA1A~PUA4A. The thermal properties of PUA1A~PUA4A were investigated by using thermal gravimetric analysis (TGA) (shown in Table 1 and Figure 7). The results showed that all samples were thermally stable under a nitrogen atmosphere of up to 215 °C. The samples of PUA1A~PUA3A underwent a two-step thermal decomposition. The weight loss at 200–310 °C is mainly due to the breaking and decomposition of the OC(O)NH bond in polyurethane molecules. The urethane bond is known to be relatively thermally unstable to decompose into amines, olefins and carbon dioxide [43]. The weight loss at 370~480 °C is due to the decomposition of the aliphatic chain. The maximum decomposition temperature of PUA4A (308 °C) is higher than PUA1A~PUA3A. This difference in thermal stability may be due to the fact that UA1~UA3 are mono acrylate urethane. In the PUA1A~PUA3A cross-linking network, the carbamate fragments are side chains with high mobility, resulting in relatively high chemical reactivity and a relatively low thermal decomposition temperature. However, in the PUA4A cross-linking network, the carbamate group is located at the backbone, which has lower mobility and higher thermal stability. The volume shrinkage of PUA1A~PUA4A is close (7.5~8.3%) and the gel content of PUA1A~PUA3A is above 85%.

**Figure 8:**
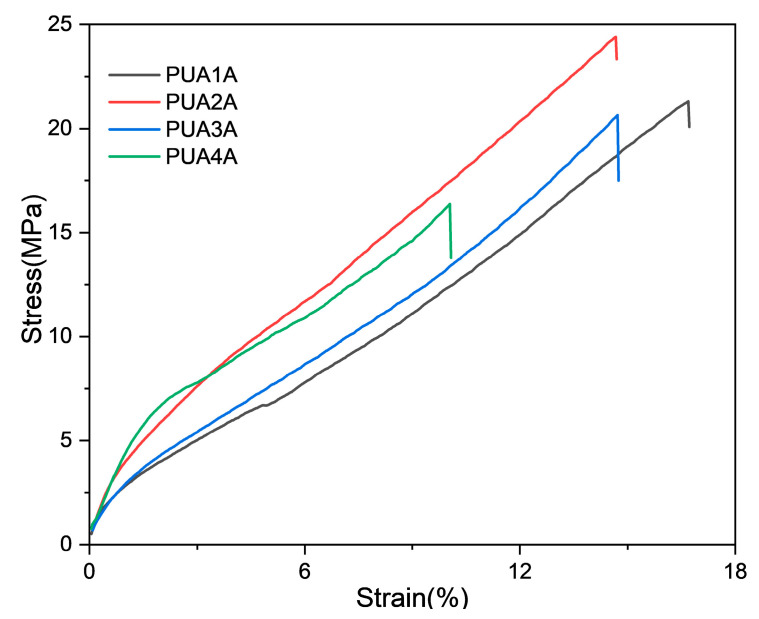
Stress–strain curves of PUA1A~PUA4A.

**Figure 9:**
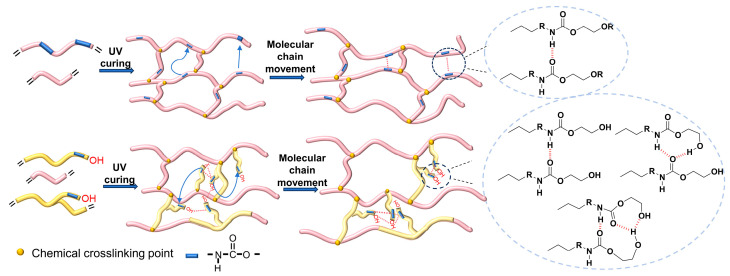
Schematic of dynamic hydrogen bond formation of PUA network.

The tensile testing of PUA1A~PUA4A was conducted, and the stress–strain curves are shown in Figure 8. The tensile data of PUA1A~PUA4A are listed in Table 1. Since UA4 is a bifunctional polyurethane acrylate which forms a higher cross-linking density after UV curing, the elastic modulus of PUA4A is greater than that of PUA1A~PUA3A. Meanwhile, the tensile strength and elongation at break of PUA4A are lower than that of PUA1A~PUA3A due to the main components in UA1~UA3, which contain some amount of mono functional urethane acrylate. Compared with UA2 and UA3, UA1 contains a higher amount of monofunctional urethane acrylate IPDA-EC-NPGDA and a lower amount of NPGDA, which results in a lower cross-linking density, elastic modulus and tensile strength than those of UA2 and UA3. The elongation at break of UA1 is larger than those of UA2 and UA3. For a higher content of NPGDA in UA2 and UA3, UA2 contains more monofunctional urethane acrylate IPDA-EC-NPGDA, which can produce a unique cross-linking network with more side chains with carbamate fragments after UV curing. The main factors leading to the difference in the mechanical properties of PUA1A~PUA4A are the cross-linking density and intermolecular forces, especially hydrogen bonding. The hydroxyl and urethane bonds on the PUA1A~PUA3A side chain have high mobility, so they can easily form stronger dynamic hydrogen bonds during the stretching process, thereby enhancing the toughness of the cured material. The hydrogen bonding model of a cross-linked network is proposed as shown Figure 9.

### 2.4. 3D Printing of PUA

**Figure 10:**
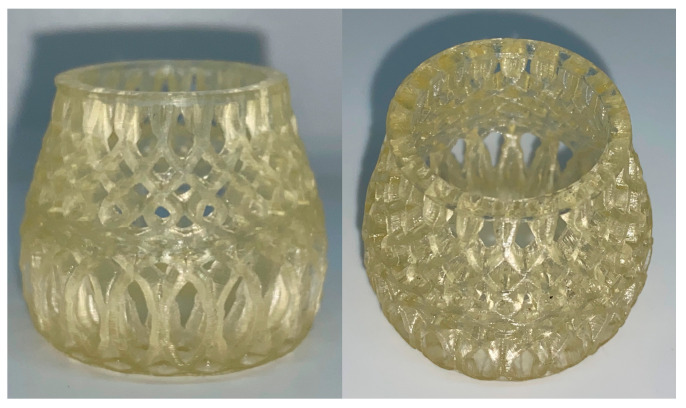
3D printed sample.

Given that UA1A has the lowest viscosity and volume shrinkage as well as a good UV curing rate, UA1 was chosen as a component for 3D printing. A UV curable mixture containing 50 wt% of urethane acrylate, 37 wt% of NPGDA, 10 wt% of ACMO, 1.5 wt% of Da-1173 and 1.5 wt% of ITX was obtained by mixing a certain amount of UA1, NPGDA, Da-1173 and ITX for 3D printing. The finished product after printing is shown in Figure 10, and the printed object has high precision and accuracy like the designed model.

## 3. Experimental

### 3.1. Materials and Methods

Neopentyl glycol diacrylate (NPGDA), 2-Isopropylthioxanthone (ITX), 2-hydroxy-2-methyl-1-phenylpropan-1-one (Da-1173) and Diphenyl (2,4,6-trimethylbenzoyl) phosphine oxide (TPO) were purchased from Tianjin Jiuri New Materials Chemical Co., Ltd. (Tianjin, China). Isophorone diamine (IPDA) and isophorone diisocyanate (IPDI) were purchased from Shanghai mairuier biochemical technology Co., Ltd. (Shanghai, China). Dibutyltin dilaurate (DBTDL) and ethylene carbonate (EC) were purchased from Tianjin Fuchen Chemical Reagent Factory (Tianjin, China). 4-Methoxyphenol (MEHQ) was purchased from Tianjin Komeier Chemical Reagent Co., Ltd. (Tianjin, China). 2-Hydroxyethyl methacrylate (HEMA) was purchased from the Tianjin chemical reagent factory. Triphenylphosphine (Ph_3_P) was purchased from Tianjin jieerzheng chemical industry trading Co., Ltd. (Tianjin, China). 4-Acryloylmorpholine (ACMO) was purchased from Shanghai macklin biochemical Co., Ltd. (Shanghai, China).

^1^H NMR spectra were recorded on a Bruker 400 spectrometer in CDCl_3_. ESI-HRMS was obtained on a Bruker Compact (spray voltage of 3.5 kV, capillary temperature of 200 °C, the scan range was set from 50 to 3000 *m*/*z*, nitrogen flow rate of 3 L/min, nitrogen pressure of 0.08 MPa). The FT-IR spectra and the photo-polymerization kinetics were recorded on a Bruker Tensor-27 spectrometer equipped with an LED light with a power of 170 mJ/cm^2^ at 395 nm (Shanghai Yun Tong Electronic Technology Co., Ltd. (Shanghai, China)). The curing film was cured under a UV lamp with a power density of 18.6 mW/cm^2^. All UV curing polymerization was carried out in air. Thermogravimetric analysis (TGA) was tested on Mettler Toledo 3 Instruments at a range of 30~800 °C with heating rates of 10 °C min^−1^. The 3D printed specimens were printed on a C1-LCD8.9-3D printer with a UV lamp (power 110 W, λ = 405 nm, 3 mW cm^−2^) of Xuchang dianchuang 3D Technology Co., Ltd. (Henan, China). The resolution of the printer was 50 μm. The test specimen model was configured printing from the bottom to top with each layer of 50 μm, and irradiation for 60 s for every layer. The mechanical properties (tensile strength and elongation at break) of the UV cured samples were obtained on a CMT6103 (MTS) with a crosshead speed of 3 mm/min. All samples (30 × 10 × 1 mm^3^) were placed at room temperature for 3 days to release internal stress [44,45]. Each sample was tested five times.

### 3.2. Synthesis of Urethane Acrylates

#### 3.2.1. Synthesis of Urethane Acrylates by Non-Isocyanate Method

Method 1: First, 17.61 g of EC (0.20 mol) was added in three equal portions to a flask with 34.06 g of IPDA (0.20 mol). The first two parts were added each hour at 46 °C. After 2 h, the third part was added at 70 °C, and the mixture was stirred for 2 h to obtain a colorless transparent amine-terminated urethane. Then, 106.12 g of NPGDA (0.50 mol) and 0.789 g of MEHQ (0.5 wt%) were added at 80 °C. Finally, the mixture was stirred for 13 h to obtain a colorless and transparent liquid named UA1.

Method 2: First, 26.42 g of EC (0.30 mol) was added in three equal portions to a flask with 51.09 g of IPDA (0.30 mol). The first two parts were added each hour at 46 °C. After 2 h, the third part EC, 42.45 g of NPGDA (0.20 mol) and 0.600 g of MEHQ (0.5 wt%) were added and the mixture was stirred at 50 °C for 1 h. Then, 148.57 g of NPGDA (0.70 mol) and 0.743 g of MEHQ (0.277 wt%) were added at 80 °C. The mixture was stirred at 80 °C for 4 h to obtain a colorless and transparent liquid named UA2.

Method 3: First, 8.81 g of EC (0.10 mol) was added in two equal portions to a flask with 25.55 g of IPDA (0.15 mol). The two parts were added each hour at 46 °C. After 2 h, 95.51 g of NPGDA (0.45 mol) and 0.649 g of MEHQ (0.5 wt%) were added at 80 °C. The mixture was stirred for 10 h to obtain a light yellow and transparent liquid named UA3.

#### 3.2.2. Synthesis of Urethane Acrylates by Traditional Method

First, 13.01 g of HEMA (0.10 mol) was added drop wisely to a three-necked flask with 11.11 g of IPDI (0.05 mol), 0.120 g of MEHQ (0.5 wt%) and 0.0725 g of DBTDL (0.23 mol%). The mixture was stirred at 45 °C for 1.5 h to obtain a colorless transparent solid named UA4.

In order to briefly describe the composition of UA1~UA4, the main components are listed in Table 2.

### 3.3. Formulations of UV Curing Composites

**Figure 11:**
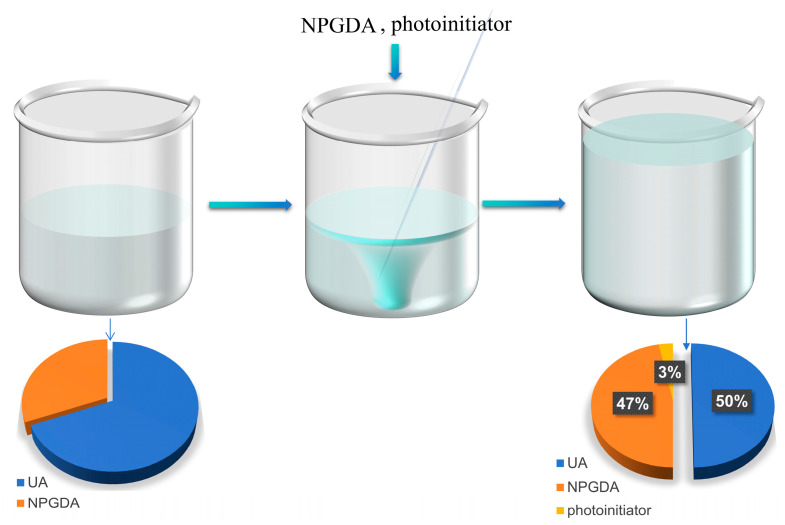
The preparation of UV curing composites.

A certain amount of NPGDA, Da-1173 and TPO were mixed with the prepared UA1~UA4 evenly to give a UV curable mixture with 47 wt% of NPGDA, 50 wt% of the urethane acrylates, 1.5 wt% of Da-1173 and 1.5 wt% of TPO named UA1A–UA4A, respectively. The preparation process is shown in Figure 11.

### 3.4. Photopolymerization Kinetics

The UV curing kinetic process of the UA1A~UA4A was explored using FT-IR. The UA1A~UA4A was uniformly coated on a KBr slice, respectively. And then FT-IR data collection and irradiation by 395 nm LED light were alternated at intervals. The conversion of the double bond was calculated by the integration of the characteristic peaks at 1722 cm^−1^ and 810 cm^−1^ according to Equation (1) [46,47]

(1) $$\mathrm{Conversion}\;\left(\%\right)=\frac{\left[{\left(A_{810}/A_{1722}\right)}_{0}-{\left(A_{810}/A_{1722}\right)}_{t}\right]}{{\left(A_{810}/A_{1722}\right)}_{0}}\times 100$$

where (A_810_/A_1722_)_0_ and (A_810_/A_1722_)_t_ represent the ratio of the integral area of the C=C double bond with the C=O double bond before and after exposure during time t, respectively.

### 3.5. Volume Shrinkage and Gel Content

After 60 s of irradiation under a mercury lamp, the 100 µm thick UA1A~UA4A coated on the I-beam coater between the two layers of release film formed a solid film. The volume shrinkage ratio of the cured film was measured by the density of the sample before and after curing. The volume shrinkage (ΔV) rate can be calculated by Equation (2)

(2) $$\Delta V\left(\%\right)=\frac{\frac{m_{0}}{\rho_{0}}-\frac{m_{0}}{\rho_{1}}}{\frac{m_{0}}{\rho_{0}}}\times 100\%$$

where m_0_ represents the mass of the sample and ρ_0_ and ρ_1_ represent the sample density before and after curing, respectively. The cured film was extracted by boiling CH_2_Cl_2_ for 12 h, then the residual solid polymer was dried at 60 °C for 12 h until a constant weight. The gel content was calculated by the following Equation (3)

(3) $$\mathrm{Gel}\;\mathrm{content}\left(\%\right)=\frac{m_{2}}{m_{1}}\times 100$$

where m_1_ and m_2_ are the mass of the film before and after being extracted by CH_2_Cl_2,_ respectively.

## 4. Conclusions

Non-isocyanate polyurethane acrylates were prepared by the reaction of diamine with carbonate, and subsequently the aza Michael addition of amine-terminated urethane with acrylate. Monofunctional acrylates containing polyurethanes can be copolymerized under UV irradiation and form a unique cross-linked network with urethane fragments as side chains. Side-chain urethane fragments are more likely to form dynamic intramolecular and intermolecular hydrogen bonds, giving the material better tensile strength (21 MPa) and elongation at break (16%). The main factors leading to the difference in the mechanical properties of PUA1A~PUA4A are the cross-linking density and intermolecular forces, especially hydrogen bonding. The novel urethane acrylates are expected to be used in the field of functional UV curing materials such as 3D printing.

## Figures and Tables

**Table 1:** Cured film properties of PUA1A~PUA4A.

Sample	T_5%_ (°C)	Modulus of Elasticity (MPa)	Tensile Strength (MPa)	Elongation at Break (%)
PUA1A	222	97.0 ± 4.2	21.3 ± 0.8	16.7 ± 1.9
PUA2A	215	184.4 ± 5.0	24.4 ± 0.8	14.7 ± 0.5
PUA3A	216	107.9 ± 8.8	20.7 ± 2.1	14.8 ± 0.6
PUA4A	311	416.2 ± 13.6	16.4 ± 1.3	10.8 ± 0.6

**Table 2:** The composition of UA1~UA4.

Sample	The Main Composition
UA1	IPDA-EC-NPGDA, IPDA-EC-2NPGDA, IPDA-2NPGDA, IPDA-3NPGDA, NPGDA
UA2	NPGDA, IPDA-EC-NPGDA, IPDA-2NPGDA
UA3	NPGDA, IPDA-2NPGDA, IPDA-EC-NPGDA
UA4	IPDI-2HEMA

## Data Availability

The data presented in this study are available on request from authors.

## Supplementary Materials

The following supporting information can be downloaded at: https://www.mdpi.com/article/10.3390/molecules29112639/s1, Figure S1: ESI-HRMS spectrum of reaction products of IPDA with EC (mole ratio of 1:2); Figure S2: ESI-HRMS spectra of UA5; Figure S3: ^1^H NMR spectrum of reaction products of IPDA with EC (mole ratio of 1:2); Figure S4: ^1^H NMR spectrum of UA2; Figure S5: ^1^H NMR spectrum of UA3; Figure S6: The FT-IR of photopolymerization kinetics. (a) UA1A; (b) UA2A; (c) UA3A; (d) UA4A; Scheme S1: The structural formula of reaction products of IPDA with EC (mole ratio of 1:2); Scheme S2: The structural formula of the product in UA5; Scheme S3: Plausible formation pathway of PEG like acrylates (* and **).
