# Supplementary material for: Non-Isocyanate Urethane Acrylate Derived from Isophorone Diamine: Synthesis, Characterization and Its Application in 3D Printing
Source: Molecules. 2024 Jun 3;29(11):2639. doi: 10.3390/molecules29112639 (PMC11173429; doi:10.3390/molecules29112639)
Supplement: Supplementary file 1 [file molecules-29-02639-s001.zip › molecules-3028638-supplementary.pdf]

Non-Isocyanate Urethane Acrylate Derived from Isophorone Diamine: Synthesis, Characterization and Its Application in 3D Printing

Xinqi Zhang [1], Xinxin Zan [1], Jiangdi Yin [1] and Jiaxi Wang [1,2,*]

[1] School of Chemical Engineering and Technology, Hebei University of Technology, Tianjin 300130, China; zxq19692021@163.com (X.Z.); 15716856616@163.com (X.Z.); 18332768376@163.com (J.Y.)
[2] Hebei Provincial Key Lab of Green Chemical Technology and High Efficient Energy Saving, Hebei University of Technology, Tianjin 300130, China
* Correspondence: wangjiaxi@hebut.edu.cn

Synthesis of IPDA-2EC and its Reaction with Acrylate

17.61 g EC (0.20 mol) and 17.03 g IPDA (0.10 mol) were added to a flask and stirred at 110 °C for 8.5 h to obtain a light yellow solid. Then 93.38 g NPGDA (0.44 mol), 0.26 g Ph_3P (1 mol%) and 0.64 g MEHQ (0.5 wt%) were added and stirred at 100 °C for 7 h to obtain a yellow liquid, named as UA5. The ESI-HRMS spectra of reaction products of IPDA with EC (mole ratio of 1:2) and UA5 were shown in Figure S1 and Figure S2, respectively. The structural formula of the products in HCT and UA5 were shown in Scheme S1 and Scheme S2.

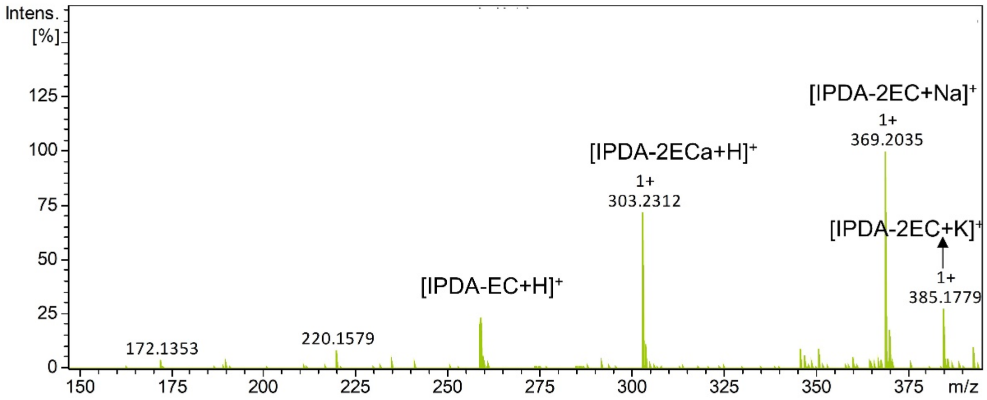

Figure S1. ESI-HRMS spectrum of reaction products of IPDA with EC (mole ratio of 1:2).

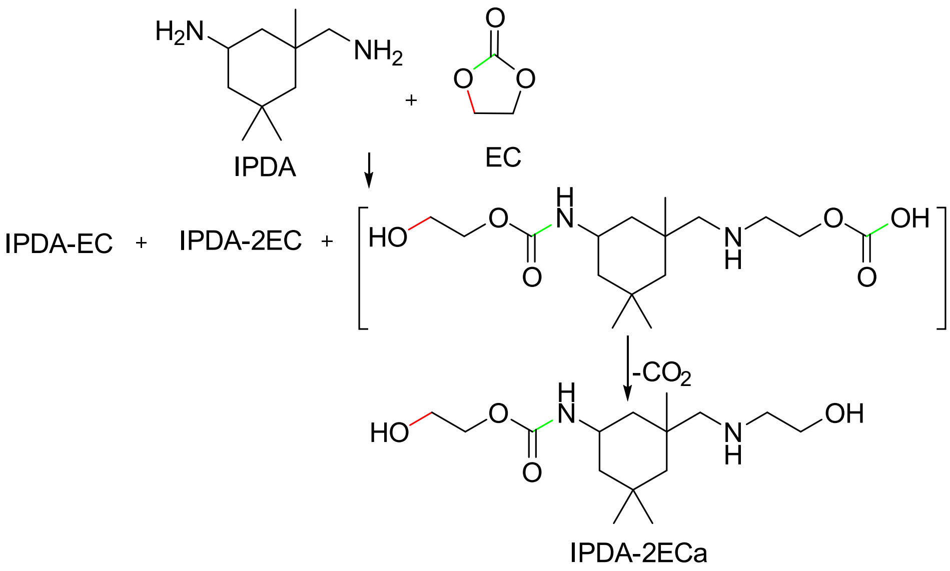

Scheme 1. The structural formula of reaction products of IPDA with EC (mole ratio of 1:2).

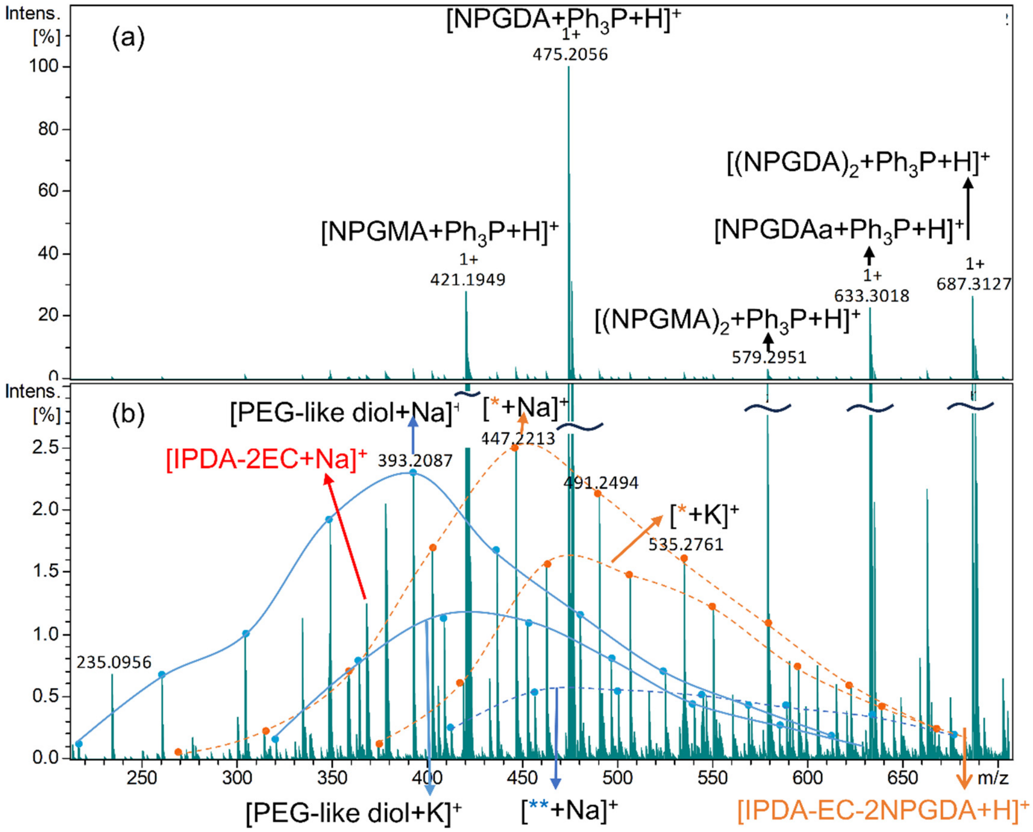

Figure S2. ESI-HRMS spectra of UA5. (**a**) Regular graph; (**b**) Enhanced graph

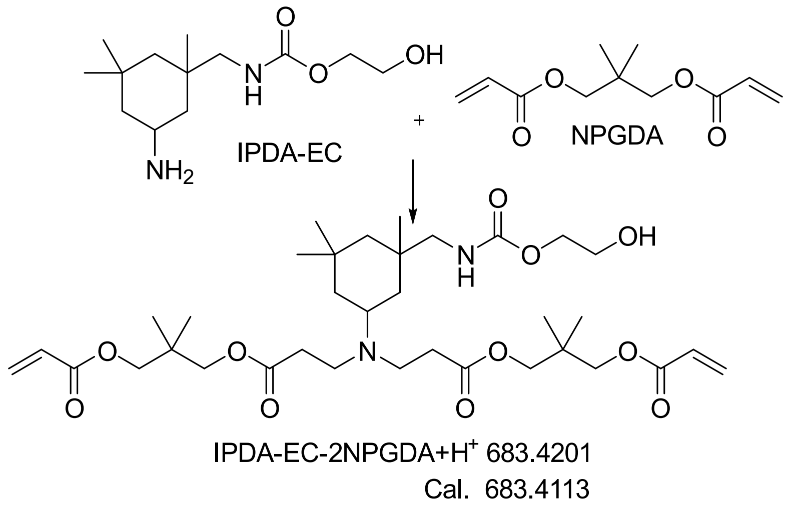

Scheme 2. The structural formula of the product in UA5.

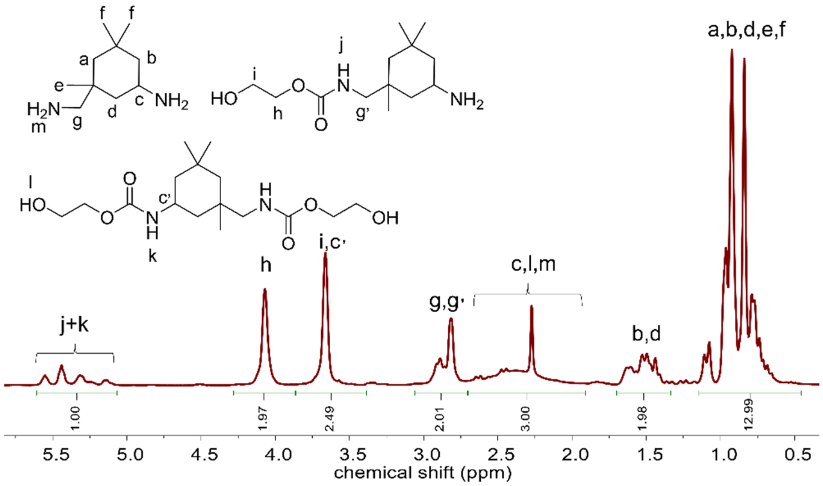

Figure S3. ^{1}H NMR spectrum of reaction products of IPDA with EC (mole ratio of 1:2).

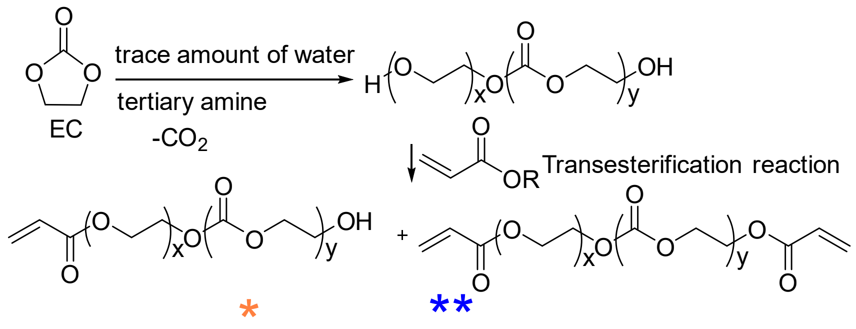

Scheme 3. Plausible formation pathway of PEG like acrylates (* and **).

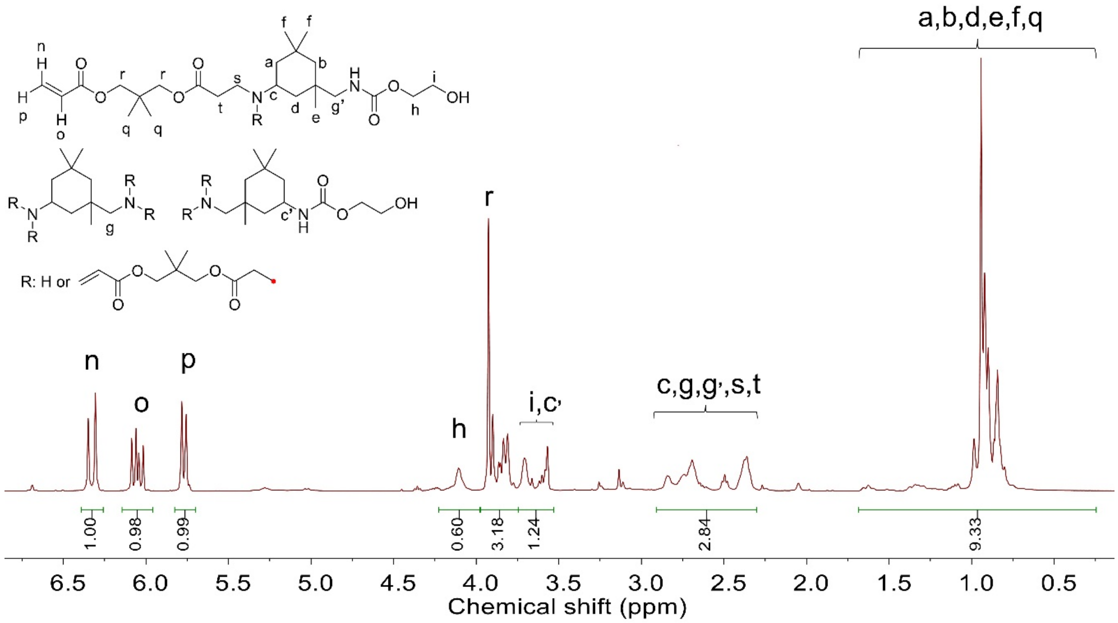

Figure S4. ^{1}H NMR spectrum of UA2.

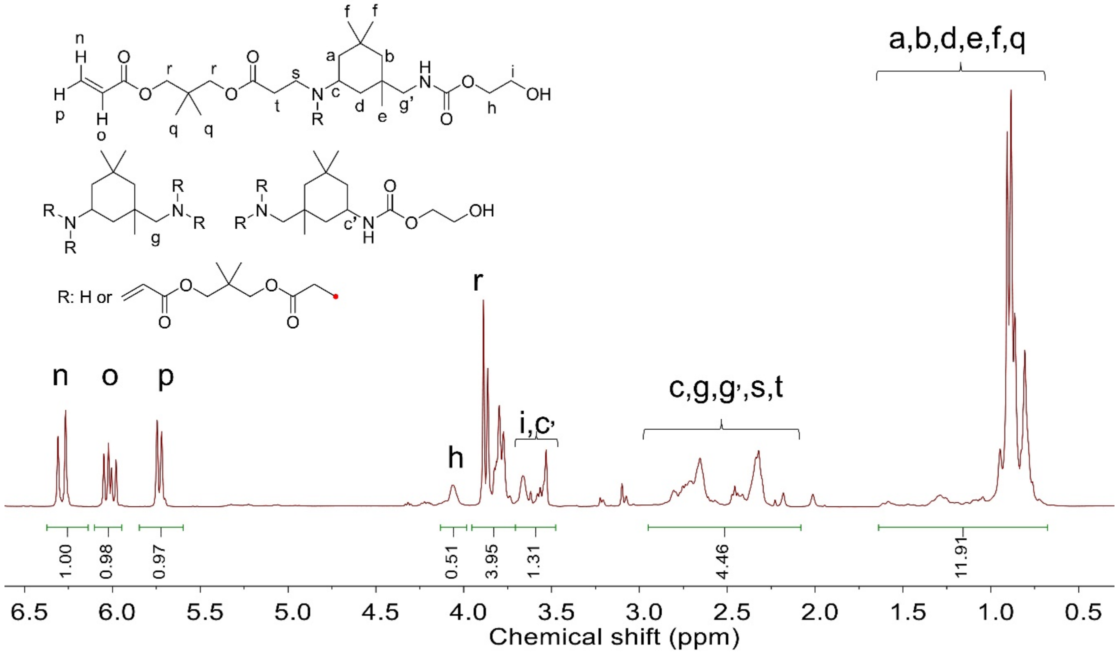

Figure S5. ^{1}H NMR spectrum of UA3.

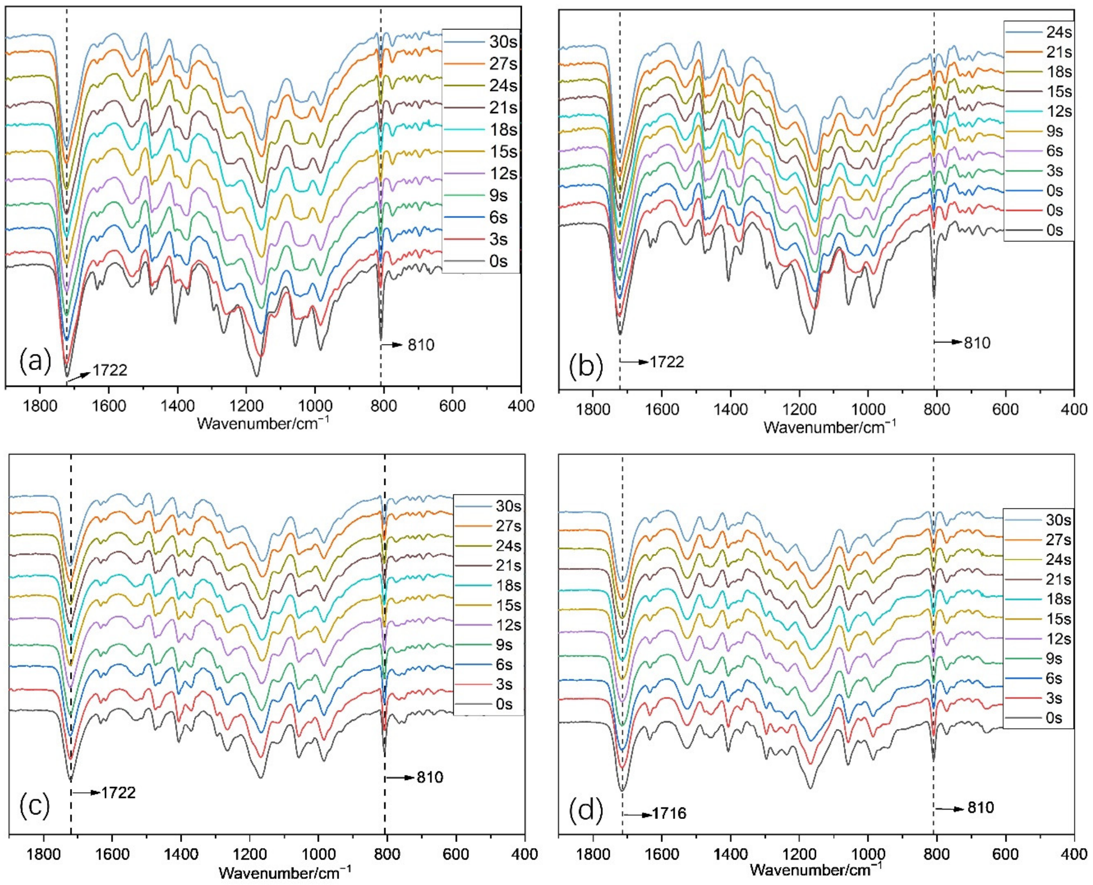

Figure S6. The FT-IR of photopolymerization kinetics. (**a**) UA1A; (**b**) UA2A; (**c**) UA3A; (**d**) UA4A
